# Prime-Boost Vaccine Regimen for SjTPI and SjC23 Schistosome Vaccines, Increases Efficacy in Water Buffalo in a Field Trial in China

**DOI:** 10.3389/fimmu.2019.00284

**Published:** 2019-02-20

**Authors:** Akram A. Da'Dara, Changlin Li, Xinling Yu, Mao Zheng, Jie Zhou, Lisa M. Shollenberger, Yue-sheng Li, Donald A. Harn

**Affiliations:** ^1^Department of Infectious Diseases and Global Health, Tufts Cummings School of Veterinary Medicine, Tufts University, Grafton, MA, United States; ^2^Department of Infectious Diseases and Center for Tropical and Emerging Global Diseases, College of Veterinary Medicine, University of Georgia, Athens, GA, United States; ^3^Hunan Institute of Parasitic Diseases, World Health Organisation Collaborating Centre for Research and Control of Schistosomiasis in Lake Region, Yueyang, China; ^4^Department of Biological Sciences, Old Dominion University, Norfolk, VA, United States; ^5^Molecular Parasitology Laboratory, Infectious Diseases Division, QIMR Berghofer Medical Research Institute, Brisbane, QLD, Australia

**Keywords:** *Schistosoma japonicum*, DNA vaccine, SjTPI, SjC23, heterologous prime-boost, water buffalo

## Abstract

Schistosomiasis remains a serious zoonotic disease in China and the Philippines. Water buffalo and cattle account for the majority of transmission. Vaccination of water buffalo is considered a key strategy to reduce disease prevalence. Previously, we showed that vaccination of water buffalo with SjC23 or SjCTPI plasmid DNA vaccines, induced 50% efficacy to challenge infection. Here, we evaluated several parameters to determine if we can develop a two dose vaccine that maintains the efficacy of the three dose vaccine. We performed four trials evaluating: (1) lab produced vs. GLP grade vaccines, (2) varying the time between prime and boost, (3) the influence of an IL-12 adjuvant, and (4) a two dose heterologous (DNA-protein) prime-boost. We found the source of the DNA vaccines did not matter, nor did increasing the interval between prime and boost. Elimination of the IL-12 plasmid lowered homologous DNA-DNA vaccine efficacy. A major finding was that the heterologous prime boost improved vaccine efficacy, with the prime-boost regimen incorporating both antigens providing a 55% reduction in adult worms and 53% reduction in liver eggs. Vaccinated buffalo produced vaccine-specific antibody responses. These trials suggest that highly effective vaccination against schistosomes can be achieved using a two dose regimen. No adjuvants were used with the protein boost, and the potential that addition of adjuvant to the protein boost to further increase efficacy should be evaluated. These results suggest that use of these two schistosome vaccines can be part of an integrated control strategy to reduce transmission of schistosomiasis in Asia.

## Introduction

Schistosomiasis continues to be a serious public health problem worldwide, with more than 200 million people infected and with an estimated 700 million people in 74 countries at risk of infection (1). Three species, *Schistosoma japonicum, S. mansoni*, and *S. haematobium*, cause the majority of disease. *S. japonicum* is the causative agent of schistosomiasis in China, the Philippines and other regions of southeast Asia (1). Unlike *S. mansoni* and *S. haematobium, S. japonicum* is a zoonotic disease (2–5). Epidemiological studies have shown that bovines, particularly water buffalo play a major role in the transmission of schistosomiasis in China and the Philippines (6, 7). Despite more than 50 years of intensive control efforts, including the World Bank Schistosomiasis Control Project from 1992 to 2001, schistosomiasis remains a major public health concern in these regions, with over one million Chinese currently infected and another 40 million living in areas at risk of infection (4, 8). The majority (>80%) of schistosomiasis cases occur around the Dongting and Poyang lakes and the marshland regions of Hunan, Jiangxi, Anhui, Hubei, and Jiangsu Provinces of China, where elimination of transmission has proved difficult (9–12).

Schistosomiasis control in China includes simultaneous praziquantel (PZQ) treatment of humans and water buffalo and reducing the number of water buffalo in endemic areas by replacing them with cattle or motorized tractors (13, 14). These control measures are time consuming, expensive, and for praziquantel treatment, recurring annually. A more sustainable option would be development of an integrated control program that, in addition to praziquantel treatment, adds vaccination of water buffalo and cattle to further reduce transmission of *S. japonicum* from bovines, potentially leading to long-term sustainable control of schistosomiasis (7, 15–17). It is important to vaccinate cattle in endemic settings as they are more susceptible to schistosome infection than water buffalo (18). In this regard, a mathematical model of schistosome transmission predicts that schistosome vaccines capable of reducing schistosome fecal egg output from water buffalo/cattle by 40% will lead to a significant reduction in transmission of schistosomiasis (3, 16).

Efficacy of the SjTPI and SjC23 plasmid DNA vaccines in water buffalo was previously shown to be 50% when administered three times using an IL-12 plasmid DNA adjuvant (16, 19). In the current study, our goal was to evaluate GLP quality plasmid DNA vs. lab-produced plasmid DNA and to reduce the vaccine regimen from three doses to two coincident with increased efficacy for these two vaccines. We found vaccine efficacy of plasmid DNA vaccines to be the same independent of the source of the plasmid DNAs. We found that extending the time between prime and boost did not change levels of plasmid DNA vaccine efficacy. We did note the positive influence on vaccine efficacy when a pIL-12 plasmid DNA was used with the pSjC23. Importantly, we observed that the two-dose, heterologous prime-boost regimen induced the highest levels of efficacy we have seen in water buffalo trials. Here, the rec SjC23 and SjCTPI proteins were administered in saline without adjuvant, suggesting that additional vaccine trials in buffalo should be performed to ascertain whether a recombinant protein homologous prime-boost vaccine regimen with adjuvant will yield significantly higher levels of efficacy than reported here.

## Materials and Methods

### Buffalo and Trial Site

Water Buffalo were purchased from Huilong county, Hunan province, from an area with no history of schistosomiasis transmission, and transported to the field site. Age, sex and weight of the different water buffaloes used in this study are summarized in the Supplementary Tables 1–4. Upon arrival at the field site, the buffalo were quarantined for 4 weeks, confirmed schistosome-free by the miracidial hatching test and ELISA, and treated with levamisole to eliminate other gastrointestinal geohelminths, as described (16). Water buffalo were then tagged with an identification number on the collar, the right ear, and into one of the horns, then divided randomly into different trial groups such that buffalo in each trial cohort had similar numbers of male and female buffalo and were of similar age and weight (Supplementary Table 1). The study site selected for the vaccine trials was Huaqiao village, Linxiang city, Hunan province.

### DNA Vaccines and Recombinant Proteins

All DNA vaccine constructs were constructed as described in Da'dara et al. (16). GLP standard plasmids (SjC23-Hsp70 and SjTPI-Hsp70) including control plasmid pVAX1, and pUMVC3-hIL-12 plasmid DNA encoding human IL-12 (pIL-12) were manufactured by Aldevron (Fargo, ND). Recombinant SjTPI (rSjTPI) and SjC23 (rSjC23) were purified using similar protocols as previously described (16). Briefly, for SjC23, the large extracellular hydrophilic domain (amino acids 108–183) of SjC23 (SjC23-loop) was expressed in *Escherichia coli* using the prokaryotic expression plasmid pET32b (Novagen, Madison, WI, USA). For SjCTPI, the pTrcHisB plasmid (Invitrogen) was used to produce full-length recombinant SjCTPI. Recombinant SjC23 and SjCTPI proteins were purified using His-Trap HP columns (GE Healthcare, Piscataway, NJ, USA). Purified recombinant proteins were analyzed by SDS-PAGE and Western blotting.

### Vaccine Study Design, Vaccination, and Challenge Infection

**Figure 1:**
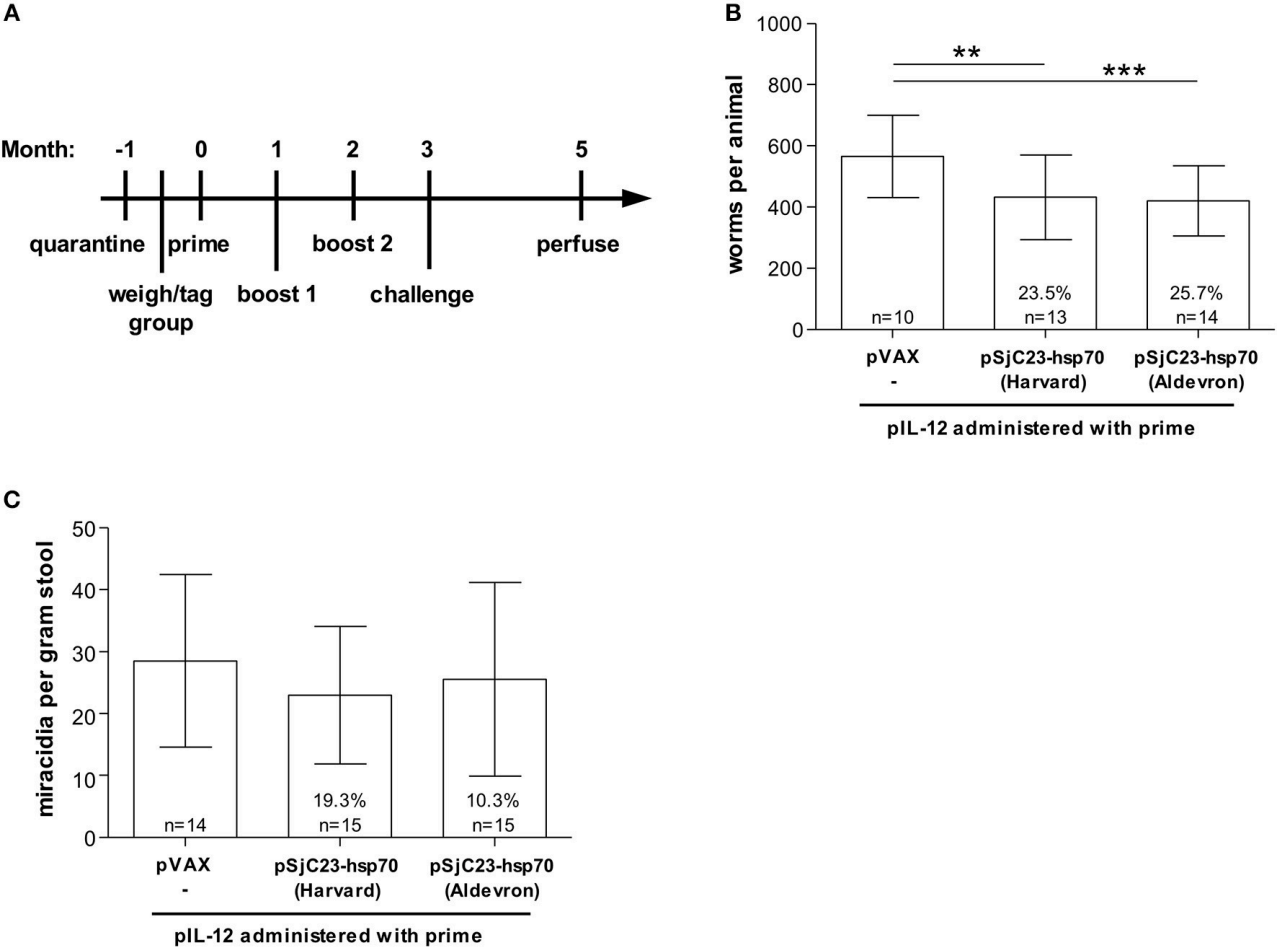
Evaluation of commercially produced, GLP grade DNA vaccine pSjC23-hsp70. **(A)** Buffalo were vaccinated with saline or pSjC23-hsp70 plasmid produced by Harvard or Aldevron three times at 1 month intervals. All animals were also given pIL-12 adjuvant plasmid at the first (prime) vaccination. One month after the third vaccination, buffalo were challenged with 1,000 cercariae. Two months post challenge, buffalo were perfused. **(B)** Worm and **(C)** fecal miracidia burdens were measured. The average and standard deviations are graphed. Percent reductions and total animals per group are listed within each bar. **p < 0.02, ***p < 0.01 by one-way ANOVA with Turkey's post-test.

Initially, we compared immunogenicity and efficacy of in-lab produced SjC23 plasmid DNA vaccines to commercially produced, GLP grade, SjC23 plasmid DNA vaccines. The trial was a randomized double-blinded study, comprised of 45 buffalo divided randomly into three groups. Plasmid DNAs were coded by an independent laboratory at the Harvard School of Public Health (HSPH), then shipped to China. Animals were immunized intramuscularly with 1 ml of 300 μg of schistosome-DNA or control pVAX vaccine plus 300 μg of IL-12 plasmid DNA for priming (Figure 1). One month after the last immunization, water buffalo were challenged with 1,000 freshly shed cercariae kindly provided by Jiangsu Institute of Parasitic Diseases, China. Eight weeks post-challenge, water buffalo were euthanized and adult worm burdens determined by perfusion. Additionally, when possible, identical portions of liver lobes were obtained to determine liver egg burdens, and fresh fecal samples were obtained to determine fecal egg counts and miracidial hatching rates as previously described (16).

**Figure 2:**
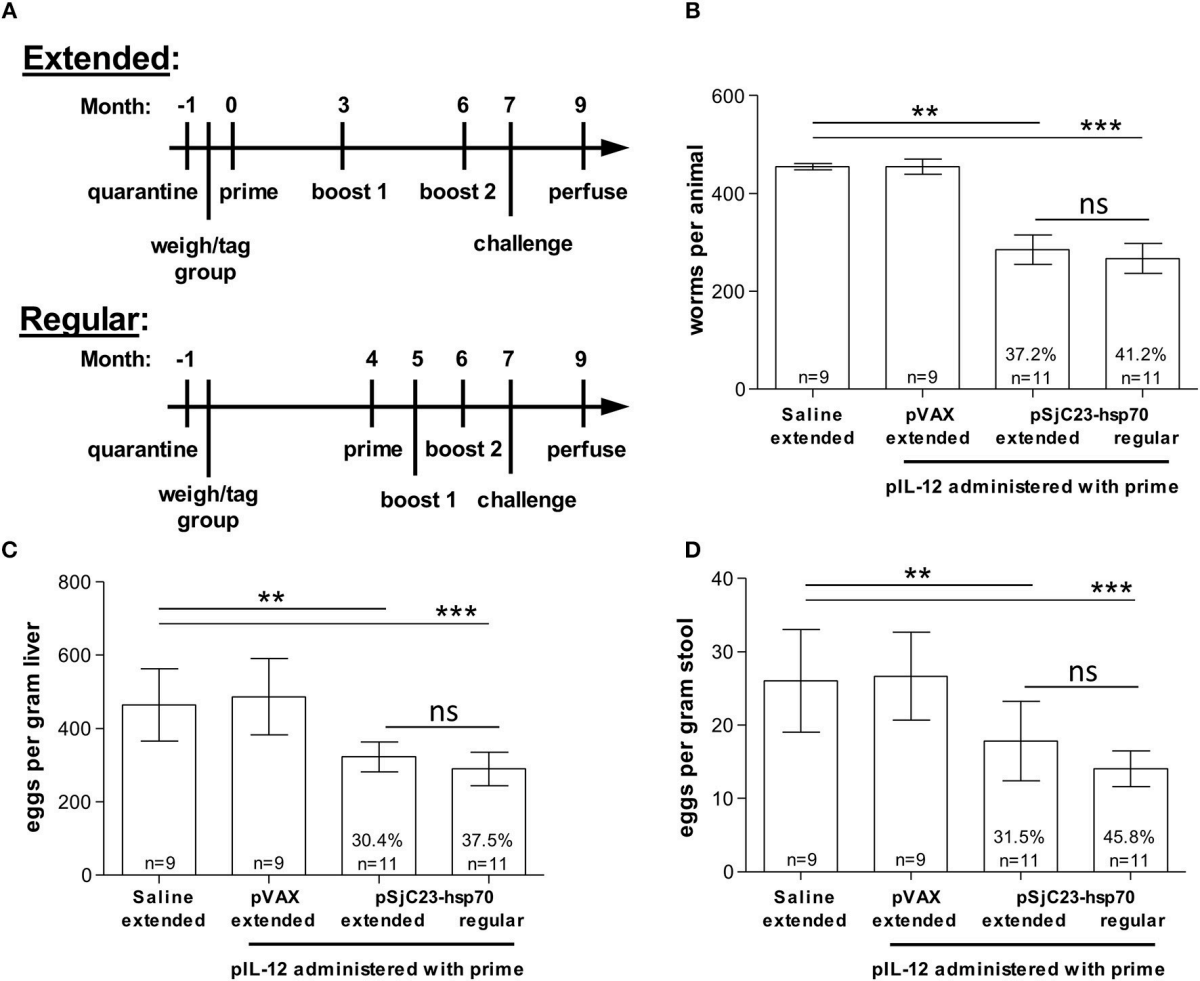
Comparison of inter-vaccination interval length. **(A)** Buffalo were vaccinated with saline, pVAX control or pSjC23-hsp70 plasmid three times at 1 month (**Regular**) or 3 month (**Extended**) intervals. All groups except for the saline injection were also given pIL-12 adjuvant plasmid at the first (prime) vaccination. One month after the third vaccination, buffalo were challenged with 800 cercariae. Two months post challenge, buffalo were perfused. **(B)** Worm, **(C)** liver egg, and **(D)** fecal miracidia burdens were measured. The average and standard deviations are graphed. Percent reductions and total animals per group are listed within each bar. **p < 0.01, ***p < 0.001 by one-way ANOVA with Turkey's post-test.

The second goal of this study was to determine if extending the time between prime and boost would improve DNA vaccine efficacy. Animals were immunized intramuscularly with schistosome-DNA or control DNA vaccines as previously described (Figure 2A). We compared 1- and 3-month intervals between prime and boost. One month after the last immunization, water buffalo were challenged with 800 cercariae freshly shed from native/local, infected snails.

**Figure 3:**
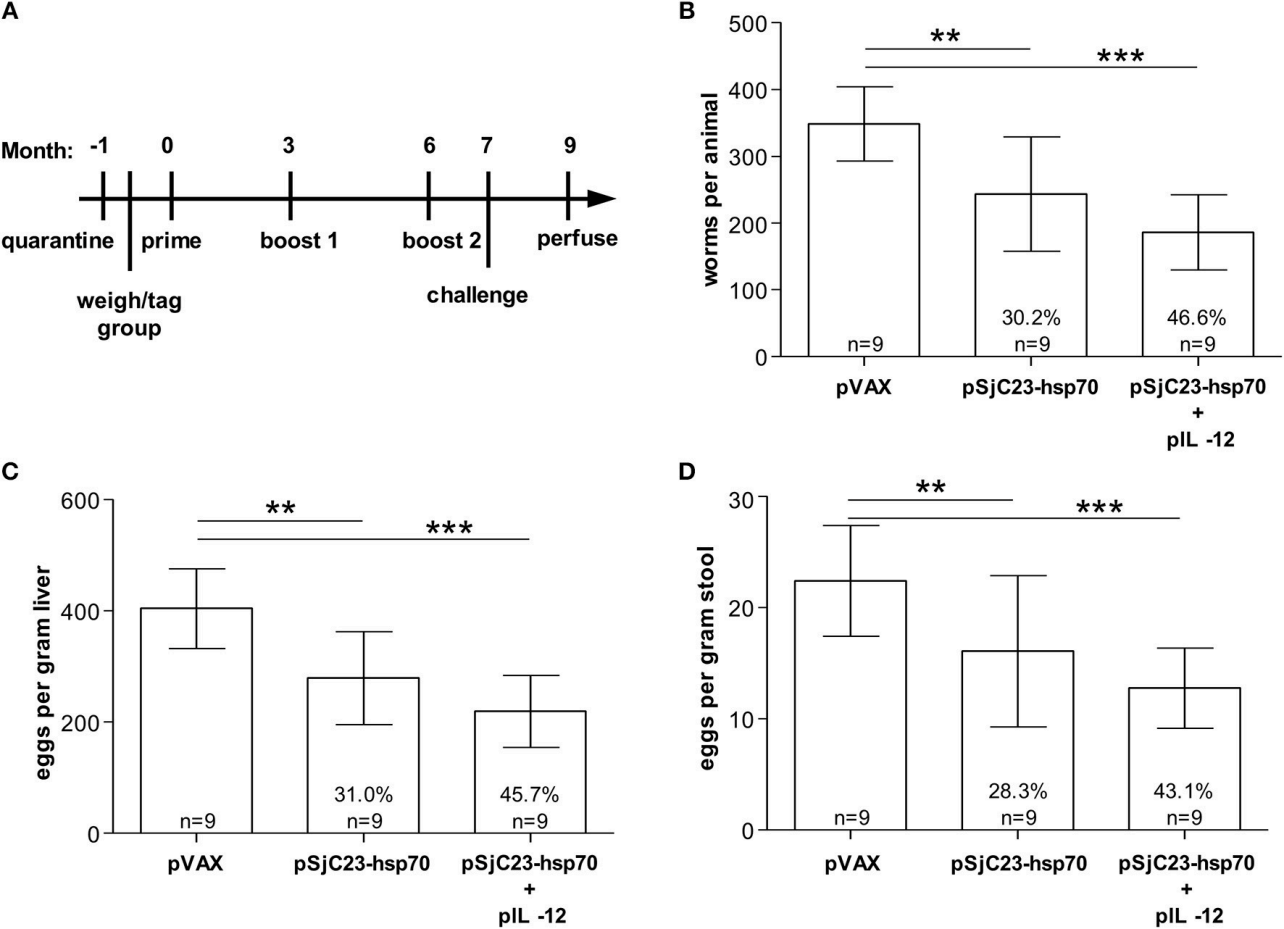
Determination of pIL-12 adjuvant requirement. **(A)** Buffalo were vaccinated with pVAX control or pSjC23-hsp70 plasmid three times at 3 month intervals. Half of the animals receiving pSjC23-hsp70 were also given pIL-12 adjuvant plasmid at the first (prime) vaccination. One month after the third vaccination, buffalo were challenged with 800 cercariae. Two months post challenge, buffalo were perfused. **(B)** Worm, **(C)** liver egg, and **(D)** fecal miracidia burdens were measured. The average and standard deviations are graphed. Percent reductions and total animals per group are listed within each bar. **p < 0.01, ***p < 0.001 by one-way ANOVA with Turkey's post-test.

We then examined the importance of the IL-12 plasmid DNA for induction of optimal levels of efficacy. Buffalo were immunized as previously described with schistosome-DNA or control DNA vaccines with or without the pUMVC3-hIL-12 plasmid DNA (Figure 3). The pUMVC3-hIL12 was administered at the prime only. One month after the last immunization, water buffalo were challenged with 800 cercariae.

**Figure 4:**
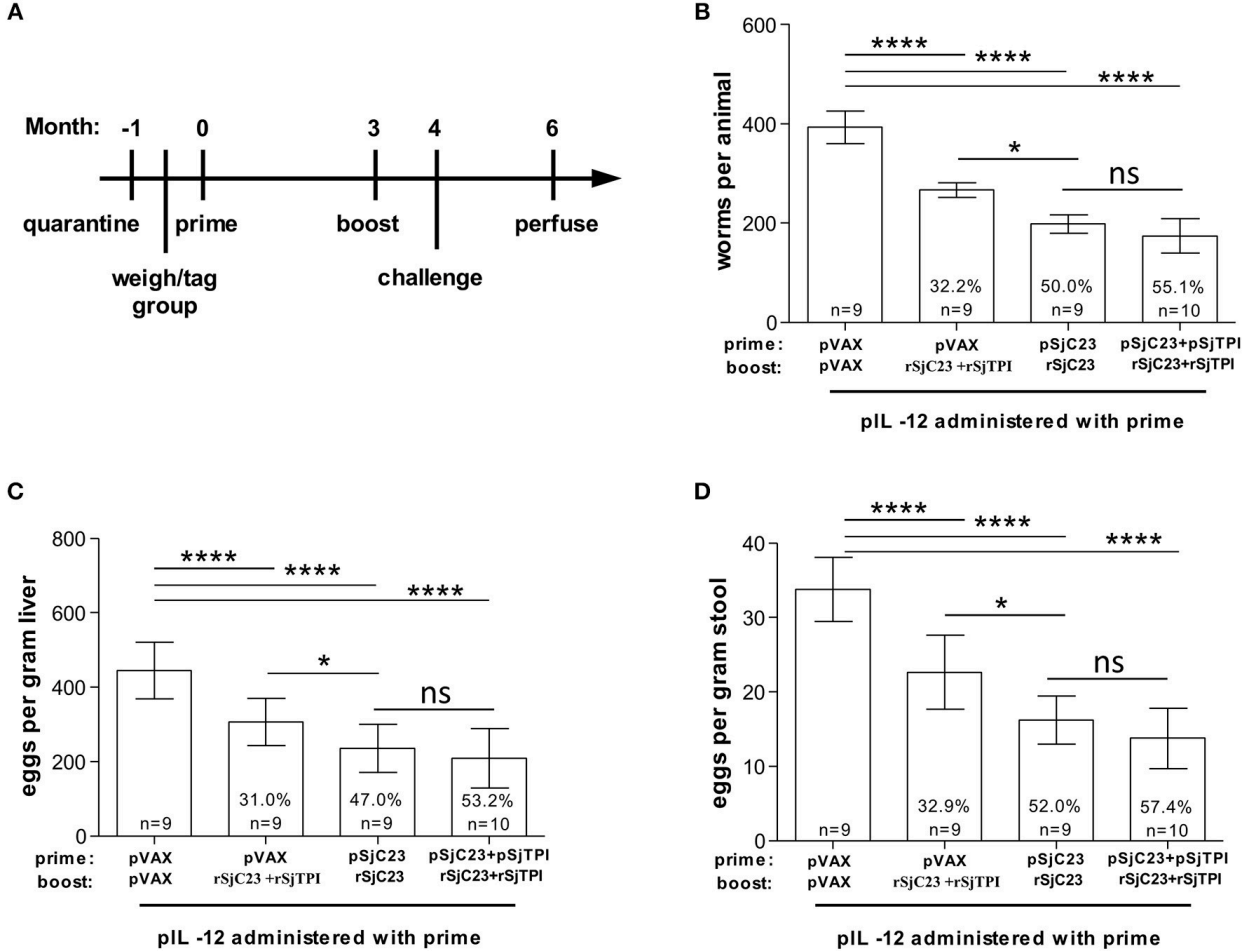
Assessment of heterologous vaccination regimen. **(A)** Buffalo were vaccinated with a DNA prime, followed by a recombinant protein boost 3 months later, as indicated. All animals were also given pIL-12 adjuvant plasmid at the first (prime) vaccination. One month after the booster vaccination, buffalo were challenged with 1,000 cercariae. Two months post challenge, buffalo were perfused. **(B)** Worm, **(C)** liver egg, and **(D)** fecal miracidia burdens were measured. The average and standard deviations are graphed. Percent reductions and total animals per group are listed within each bar. *p < 0.05, ****p < 0.0001 by one-way ANOVA with Turkey's post-test.

Finally, we evaluated efficacy of a heterologous plasmid DNA prime with a recombinant protein boost. In this trial, we employed combination SjTPI/SjC23 vaccines. Buffalo were primed as described with 300 μg of both the SjC23 and SjTPI DNA vaccines or control DNA vaccine. Booster vaccination was with plasmid DNAs or with 100 μg each, recombinant SjC23 and SjTPI proteins (Figure 4A). One control group received control pVAX DNA as prime and were then boosted with recombinant antigens. One month after the last immunization, water buffalo were challenged with 1,000 freshly shed cercariae.

### Water Buffalo Perfusion and Analysis of Worm Burden

Two months post-challenge, water buffalo were weighed then euthanized. Adult worms were obtained from the portal vein by perfusion of the descending thoracic aorta with physiologic saline. Worms trapped in intestinal tissue or caught up in tissues near where the portal vein was cut, were collected by hand and counted. All adult worms recovered from each water buffalo were counted and recorded as total worm burdens.

### *S. japonicum* Eggs in Liver

Two samples from the left lobe and one sample from the right lobe were taken from the livers of each water buffalo and weighed. Each piece was placed in 20 ml of 4% (w/v) KOH for 2 days at 37°C. The suspension was then agitated to re-suspend the mixture, 1 ml of the liver suspension was collected, centrifuged at 3,000 rpm for 1 min, the pellet re-suspended in 200 μl of PBS and the total eggs present were counted under a microscope. Using the average of three separate samples, the number of eggs per gram of liver tissue were calculated for each animal.

### *S. japonicum* Eggs in Stool Samples

Three fecal samples were collected from the rectum of each water buffalo 2 days and 1 day prior to perfusion and again on the day of perfusion. For each fecal sample, the number of eggs per gram (EPG) of stool was determined by microscopy and the number of hatched miracidia per gram (MPG) of stool was determined as previously described (20).

### Serological Immunoassay

Serum samples from four different buffalo from each group were randomly selected then analyzed by Western blot and ELISA to determine the antigen-specific antibody levels in sera from vaccinated buffalo as described (16).

### Statistical Analysis

GraphPad Prism version 5 was used for statistical analysis. One-way ANOVA with Tukey's post-test or two-way ANOVA and Bonferroni's post-test were used to identify the significance between various groups. Statistical significance was accepted at *p* < 0.05.

## Results

### GLP Grade DNA Vaccine Sj23-Hsp70 Has Similar Efficacy to In-lab Produced DNA Vaccine

In order to meet potential larger vaccine doses and reduce costs, we contracted with Aldevron, a leading company in the production of plasmid DNA and recombinant proteins, to produce each DNA vaccine and the control plasmid DNA under GLP conditions. To determine if GLP grade production would influence vaccine efficacy, we compared the Aldevron GLP grade vaccines to our in-house DNA vaccines using the regimen outlined in Figure 1A. Vaccination of water buffalo with the Sj23-Hsp70 construct produced at the Harvard School of Public Health or from Aldevron resulted in nearly identical levels of efficacy, 23.5 and 25.7% respectively, with no significant differences of mean adult worm burdens (*p* = 0.816) between these two vaccine groups as determined by One-ANOVA with Turkey's post-test (Figure 1B). Interestingly, we also observed lower reductions in miracidial hatching rates of 19 and 10% respectively (Figure 1C). Table 1 summarizes this data.

**Table 1:** Vaccination with pSj23-hsp70 from 2 sources.

	**Worms**			**Miracidia**		
**Group**	***n^†^***	**Total worms per animal (X ± SD)**	**Reduction and Statistics**	***n***	**Eggs per gram feces (X ± SD)**	**Reduction and Statistics**
pVAX	10	565.7 ± 134.208		14	28.506 ± 13.927	
Harvard	13	431.692 ± 137.602	23.5% *p* < 0.01	15	22.976 ± 11.112	19.3% n.s.
Aldevron	14	420.286 ± 114.603	25.7% *p* < 0.02	15	25.570 ± 15.623	10.3% n.s.

† *Several buffalo were excluded due to improper perfusion*.

*pIL-12 was administered at the prime for all groups*.

*p-value compared to pVAX by one-way ANOVA and Turkey's post-test*.

### Extending the Time Between Priming and Boosting May Reduce DNA/DNA Vaccine Efficacy

Previous studies have reported that extending the interval between priming and boosting can influence vaccine efficacy (21–24). To test this, we increased the time between prime and boost from 1 to 3 months. Using the SjC23 DNA/DNA prime boost-boost with 1 or 3-month intervals, we found that efficacy, as determined by worm burden, liver eggs/gram and fecal eggs/gram, was higher in animals where boosts were given at 1-month intervals (Table 2 and Figure 2). We observed significant reductions in adult worm burdens of 37.2 and 41.2% and reductions in eggs/gram liver of 30.4 and 37.5% for SjC23 DNA vaccinated groups at extended (3 month) or regular (1 month) intervals, respectively (Figures 2B,C). The reduction in eggs per gram stools of 31.5 and 45.8% for extended and regular month interval groups (Figure 2D). However, there was no statistically significant difference between the extended and the regular immunization regimen in all measured parameters.

**Table 2:** Comparison of time between vaccinations: 1 month (regular) or 3 months (extended).

		**Worms**		**Liver eggs**		**Miracidia**	
**Group (time between injections)**	***n***	**Total worms per animal (X ± SD)**	**Reduction and Statistics**	**Eggs per gram liver (X ± SD)**	**Reduction and Statistics**	**Eggs per gram feces (X ± SD)**	**Reduction and Statistics**
Mock (extended)	9	454.44 ± 19.42		464.11 ± 98.43		26.00 ± 7.00	
pVAX (extended)	9	454.67 ± 15.18		486.11 ± 103.99		26.67 ± 6.00	
pSjC23-Hsp70 (extended)	11	285.27 ± 30.19	37.23% *p* < 0.0001	323.00 ± 40.91	30.4% *p* < 0.0001	17.82 ± 5.40	31.46% *p* < 0.01
pSjC23-Hsp70 (regular)	11	267.27 ± 30.46	41.19% *p* < 0.0001	290.09 ± 45.68	37.5% *p* < 0.0001	14.09 ± 2.43	45.81% *p* < 0.001

*pIL-12 was administered at the prime for all groups but mock*.

*p-value compared to mock by one-way ANOVA and Turkey's post-test*.

*No significant difference between extended and regular*.

### Influence of IL-12 Plasmid DNA on DNA Vaccine Efficacy

IL-12 is an important cytokine that has the potential to activate natural killer cells and promote cytolytic T cell proliferation and has been used as an adjuvant to promote Th1-type and cellular immune responses in various DNA vaccine trials (25–29). We previously reported that administration of IL-12 with the boost enhanced host immunity and better protective efficacy in water buffalo (16, 19). However, we did not know if our DNA vaccines would retain similar protection without IL-12. The vaccination trial in water buffalo performed here showed that addition of Plasmid DNA encoding IL-12 enhanced all aspects of DNA vaccine efficacy (Table 3). Adult worm burdens were reduced by 46.6% in buffalo co-administered plasmid DNA IL-12 compared to 30.2% in buffalo vaccinated with SjC23 plasmid DNA and no IL-12 (Figure 3B). Similarly, the number of eggs/gram liver tissue was reduced 45% in buffalo co-administered plasmid DNA IL-12 compared to 31% in buffalo vaccinated with SjC23 plasmid DNA and no IL-12 (Figure 3C). A similar influence of co-administering plasmid DNA encoding IL-12 was seen in eggs/gram stool (Figure 3D). Overall, efficacy data from the DNA prime-boost experiments evaluating interval between prime and boost or value of the IL-12 plasmid were similar to what we previously reported for our plasmid DNA vaccines (16) and show that co-administration of the IL-12 encoding plasmid enhances DNA vaccine efficacy, yet not statistically significant from the no IL-12 plasmid.

**Table 3:** The role of IL-12.

		**Worms**		**Liver eggs**		**Miracidia**	
**Group**	***n***	**Total worms per animal (X ± SD)**	**Reduction (%)**	**Eggs per gram liver (X ± SD)**	**Reduction (%)**	**Eggs per gram feces (X ± SD)**	**Reduction (%)**
pVAX	9	348.56 ± 55.60		404.23 ± 72.15		22.40 ± 4.97	
pSjC23	9	243.33 ± 85.79	30.19% *p* < 0.01	279.02 ± 83.53	30.97% *p* < 0.001	16.07 ± 6.81	28.26% *p* < 0.5
pSjC23/pIL-12	9	186.00 ± 56.20	46.64% *p* < 0.0001	219.54 ± 64.75	45.69% *p* < 0.0001	12.75 ± 3.60	43.08% *p* < 0.01

*pIL-12 was only administered at the prime*.

*p-value compared to pVAX by one-way ANOVA and Turkey's post-test*.

*Addition of IL-12 encoding plasmid enhanced all vaccine efficacies, yet the difference was not statistically significant*.

### Boosting With Recombinant SjTPI and/or SjC23 Improves Vaccine Efficacy

Boosting with recombinant proteins following a DNA prime immunization elicits a broad humoral immune response and enhances the cell-mediated immune responses in mice and non-human primates (30–34). Therefore, here we moved from a three-dose plasmid DNA vaccine regimen to a two-dose plasmid DNA prime and recombinant protein boost regimen with a 3-month interval between prime and boost (Figure 4A). Groups of water buffalo were primed with pVAX control plasmid DNA, SjC23-Hsp70 plasmid DNA, or with a combination of SjC23-Hsp70 and SjTPI-Hsp70 plasmid DNAs. Three months post-prime, groups of water buffalo were boosted with recSjC23 or a combination of recSjC23 and recSjTPI (Figure 4). The results of these trials demonstrated that buffalo only receiving recSjC23 following the control pVAX prime had adult worm burdens reduced by 32.2% compared to pVAX primed and boosted buffalo (Table 4; Figure 4B). Similarly, eggs/gram liver were reduced by 31.0% in buffalo that only received the SjC23 boost following control pVAX prime (Figure 4C). Vaccine efficacy significantly increased in the two-dose regimen when buffalo were primed with SjC23-Hsp70 DNA then boosted with recSjC23. Here, adult worm burdens were reduced by 50.0% compared to controls and eggs/gram liver were reduced 47.0% compared to control vaccinated buffalo. However, we observed the highest levels of efficacy in this two-dose regimen trial when buffalo were primed with the combination of SjC23-Hsp70 and SjTPI-Hsp70 plasmid DNAs followed by boost with a combination of recSjC23 and recSjTPI. This combination yielded the highest level of vaccine efficacy that we have observed to date for these candidate vaccines in water buffalo. Adult worm burdens were reduced by 55.1% and eggs/gram liver by 53.2% compared to levels in control vaccinated buffalo (Figures 4B,C). Using DNA prime/protein boost, the reduction in worm burdens (Figure 4B), eggs in liver tissues (Figure 4C) and hatched miracidia in stool (Figure 4D) were dramatic compared to that in the control vaccinated group (*p* < 0.0001).

**Table 4:** DNA prime with recombinant protein boost.

		**Worms**		**Liver eggs**		**Miracidia**	
**Group (prime + boost)**	***n***	**Total worms per animal (X ± SD)**	**Reduction (%)**	**Eggs per gram liver (X ± SD)**	**Reduction (%)**	**Eggs per gram feces (X ± SD)**	**Reduction (%)**
pVAX + pVAX	9	393.33 ± 32.99		444.44 ± 76.01		33.78 ± 4.30	
pVAX + rSjC23	9	266.67 ±14.73	32.2% *p* < 0.0001	306.67 ± 63.25	31% *p* < 0.0001	22.67± 5.00	32.89% *p* < 0.001
pSjC23 + rSjC23	9	198.00 ± 18.74	49.66% *p* < 0.0001	235.56 ± 64.65	47% *p* < 0.0001	16.22 ± 3.23	51.98% *p* < 0.001
pSjC23/pSjTPI+ rSjC23/rSjTPI	10	176.80 ± 33.43	55.05% *p* < 0.0001	208.00 ± 74.95	53.2% *p* < 0.0001	14.40 ± 4.30	57.37% *p* < 0.001.

*All groups received pIL-12 at the prime*.

*p-value compared to pVAX by one-way ANOVA and Turkey's post-test*.

*No significant difference between the third and the fourth groups*.

**Figure 5:**
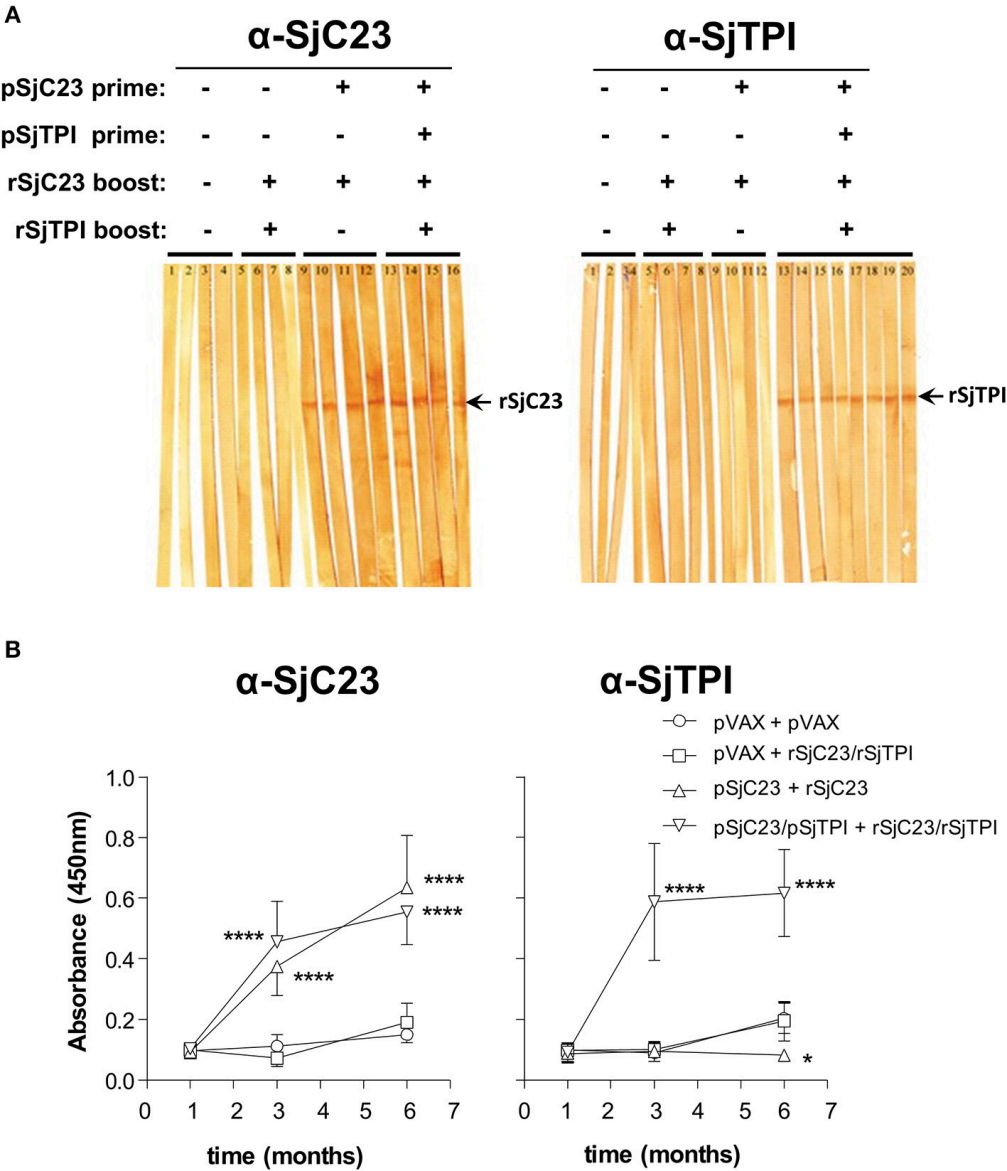
Analysis of the humoral immune response to heterologous vaccine regimen. **(A)** Western Blot analysis of individual serum samples obtained from vaccinated animals from each group prior to challenge infection. Sera from animals primed with pVAX and boosted with pVAX (lanes 1–4) or primed with pVAX and boosted with rSjC23 and rSjCTPI (lanes 5–8) did not react with rSjC23 (left panel) or rSjCTPI (right panel). Sera from animals primed with pSjC23-Hsp70 DNA vaccine and boosted with rSjC23 (lanes 9-12) elicited specific antibody responses with rSjC23, approximate MW of fusion protein is 26kDa (left panel) but not with rSjTPI, approximate MW of fusion protein is 31 kDa (right panel). Sera from animals in group 4 can elicit immune responses to both specific antigens rSjC23 (left panel, lanes 13-16) and rSjTPI (right panel, lanes 13-20). **(B)** Total IgG antibodies for all individual animals were analyzed by ELISA. Serum samples were obtained at 4 days prior to prime, 2 days prior challenge infection, and 2 days prior to perfusion. Left panel: anti-SjC23; right panel: anti-SjTPI. Values are expressed as means ± SE of the OD values of all animals within the same group. *p < 0.05, ****p < 0.0001, analyzed with two-way ANOVA.

We next evaluated sera from control and immunized animals for anti-SjC23 or SjTPI specific antibodies using Western blots and ELISA (Figure 5). We randomly picked four or more individual serum samples from each group to detect antigen-specific antibodies using Western blot. Sera from animals primed with SjC23-Hsp70 alone in in combination with SjTPI-Hsp70 then boosted with the correlate recombinant antigen elicited specific antibody responses to the respective antigens (Figure 5A). In contrast, sera from animals primed with the control pVAX plasmid and boosted with either pVAX or recombinant proteins did not react with rSjC23 (Figure 5A left panel, lanes 1-8) or rSjTPI (Figure 5A right panel, lanes 1-8). Individual sera collected at different time points from animals were used in ELISA. ELISA results showed that antibody levels are significantly increased after boosting with recombinant proteins in group 3 and 4 (Figure 5B).

## Discussion

Over the past 60 years, despite great progress in China to control *S. japonicum*, national surveys have shown that prevalence in humans in areas of endemicity did not substantially change from 1995 (4.9%) to 2004 (5.1%) (35). The current control strategy for schistosomiasis in humans and water buffalo has provided prevalence at the targeted 5% level. The targeted level was revised with the goal of reducing prevalence and infection to under 1% by 2015 (14).

Bovines (cattle and water buffalo) are the major reservoirs for *S. japonicum* infection in China and the Philippines, with estimates that 75–90% of egg contamination comes from this source. Maintaining the current praziquantel-based control strategy for bovines in the Lakes and Marsh regions of China will be difficult and costly. In addition, there is always the possibility that new endemic regions will be found in China. However, the use of integrated control strategies that incorporate a schistosome vaccine that significantly reduces passage of eggs from buffalo and cattle will aid these long-term schistosome control strategies (36–38). The level of efficacy required for the transmission blocking schistosome vaccine to help reduce prevalence of schistosomiasis has been estimated to be 45–50% reduction in fecal eggs (4). Such a vaccine could be incorporated as part of a multi-component integrated control program (39).

Our previous studies in Hunan province evaluated the efficacy of two vaccine candidates (SjTPI and SjC23) as three-dose plasmid DNA vaccines against *S. japonicum* in water buffalo (16, 19). Mathematical modeling suggests that either of these two vaccines, in combination with human chemotherapy, could lead to a significant reduction in schistosome transmission (3, 16). Here, we report the results from four recent vaccine trials performed at the same site with the goal of reducing the vaccine regimen from three to two vaccinations, coincident with increasing vaccine efficacy. Focusing on eventual large scale production of these vaccines we initially compared GLP grade plasmid DNA vaccines to lab produced plasmid DNA vaccines. GLP plasmid DNAs are better quality and a necessary step toward GMP product. Large scale production by GLP/GMP capable contractors is also less expensive than lab grade produced plasmid DNA vaccines. The results from Trial 1, shown in Figure 2, demonstrate that there is no statistical difference in vaccine efficacy in buffalo vaccinated with GLP grade SjC23 DNA vaccines vs. vaccinated with lab-produced SjC23 DNA vaccines. Thus, if plasmid DNA vaccines are to eventually be part of a vaccine regimen employing pSjC23 and/or pSjCTPI schistosome vaccines, cost-effective GMP production will benefit scale up for future vaccine trials. We were surprised by the overall low levels of protective efficacy induced by either vaccine in Trial 1. The level of efficacy observed in Trial 1 is approximately 50% lower than previously reported by us, and 50% lower than seen in Trials 2 and 3 reported in this study. The data from Trials 2 and 3 are closer to those previously reported for SjC23 DNA vaccine. We do not have an explanation as to why the level of efficacy in Trial 1 was lower than that seen in the other trials

In Trial 2 we tested for an effect of the prime-boost interval, comparing a 1 month interval to a 3 month interval. Surprisingly, increasing the interval between vaccine doses to 3 months did not increase efficacy as we hypothesized. In fact, although the differences between groups are not significant, the 1 month interval group had a trend toward higher levels of efficacy in all parameters measured as compared to the 3 month interval group, Figure 2. As expected, IL-12 as an adjuvant is proved to be important for the three-dose SjC23 DNA vaccine if protection levels of 40–50% are minimum targets of vaccine efficacy. Of note, although the use of pIL-12 plasmid during the prime increased efficacy in all parameters measured, the differences in level of efficacy between the pSjC23 vaccinated group and the pIL-12+pSjC23 groups were not significant.

The final approach to increase vaccine efficacy and reduce the regimen to two doses, was to employ a heterologous prime-boost vaccination strategy. Here plasmid DNA vaccines are used for the with recombinant protein antigens used for the boost. Heterologous prime-boost strategy has been successfully applied to many different types of diseases in animal models, including acquired immune deficiency syndrome (AIDS) (40), Japanese encephalitis virus (33) and malaria (41), and have shown greater efficacy than the homologous prime. Here we compared prime with pIL-12+SjC23, boost with recSjC23 to buffalo that were primed with pIL-12+SjC23+SjCTPI plasmid DNAs, then boosted with both recSjCTPI+recSjCTPI in saline and no adjuvant. This work led to two important observations. First, using SjC23 we demonstrated that the heterologous prime-boost, two-dose regimen induced levels of efficacy similar to that reported by us using a 3-dose plasmid DNA vaccine regimen (16, 19). Second, by employing both antigens for the plasmid DNA prime and the recombinant protein boost, we increased levels of efficacy further, resulting in a 55% reduction in adult worms and 53% reduction in eggs/gram liver compared to levels in control vaccinated buffalo. These are the highest levels of vaccine efficacy observed by us to date and this was achieved using a simple prime-boost regimen without addition of any adjuvant or delivery vehicle for the recombinant protein boost.

Several schistosome vaccines have been developed, all providing partial protection against *Schistosoma* infection. The majority of schistosome vaccines reduce adult worm burdens by <50% (42). Further, the majority of these vaccines have only been evaluated for efficacy in genetically identical inbred mice. The SjCTPI Heterologous prime-boost vaccine is currently being field-tested in bovines under natural challenge infection conditions in Samar, the Philippines as part of an integrated control package involving bovine vaccination, chemotherapy, and snail control (7). The linking of vaccination with chemotherapy would reduce overall morbidity and limit the impact of re-infection. Such a novel control program for schistosomiasis would improve significantly on the current strategy, which is based on chemotherapy alone.

Overall, our data provides considerable optimism toward the development of schistosome vaccines that are efficacious in natural, outbred hosts. Further, as these two vaccines can be further optimized by inclusion of adjuvant with the recombinant proteins, it is likely efficacy will further increase. This study also demonstrates how international team collaborations contribute to the development of effective vaccines for schistosomiasis. New antigen formulation and delivery methods of these vaccine are currently being tested in the Philippines. We believe that eventually, addition of effective schistosome vaccines as components of integrated control will be what is needed for long term control of disease.

## Ethics Statement

The study protocol was approved by the Scientific Steering Committee of the Hunan Institute of Parasitic Diseases and anti-schistosomiasis office, Department of Hunan Public Health. All animal owners were informed about the purpose and procedures of the study before being asked for their consent to participate. Permits for the described field studies were obtained from Yueyang County Animal Husbandry Bureau and Yuanjiang Municipal Animal Husbandry and Fishery Bureau.

## Author Contributions

AD designed and produced the Harvard Lab plasmid DNAs, helped design the experiments and contributed to manuscript preparation. CL produced recombinant SjC23 and SjTPI and co-wrote the manuscript. LS contributed to writing the manuscript, analyzed data, and prepared Figures and Tables. XY, MZ, and JZ performed the field vaccine trials along with YL. YL planned the experiments with DH. DH planned the experiments and co-wrote the manuscript.

### Conflict of Interest Statement

The authors declare that the research was conducted in the absence of any commercial or financial relationships that could be construed as a potential conflict of interest.

## Abbreviations

pVAX: empty plasmid DNA control vector
pSjC23-hsp70: plasmid DNA encoding C23 gene of *Schistosoma japonicum* fused to bovine heat shock protein 70
pSjC23: plasmid DNA encoding C23 gene of *Schistosoma japonicum*
pSjTPI: plasmid DNA encoding TPI gene of *Schistosoma japonicum*
rSjC23: recombinant C23 protein of *Schistosoma japonicum*
rSjTPI: recombinant TPI protein of *Schistosoma japonicum*
pIL-12: plasmid DNA encoding human interleukin 12 (IL-12)
PZQ: praziquantel
EPG: eggs per gram
MPG: miracidia per gram.
