# Supplementary material for: Prime-Boost Vaccine Regimen for SjTPI and SjC23 Schistosome Vaccines, Increases Efficacy in Water Buffalo in a Field Trial in China
Source: Front Immunol. 2019 Feb 20;10:284. doi: 10.3389/fimmu.2019.00284 (PMC6391362; doi:10.3389/fimmu.2019.00284)
Supplement: Supplementary file 1 [file Table_1.DOCX]

**Supplemental Table 1.** Characteristics of buffalo in field trial 1.

|  |  | **Sex** | |  | **Age** |  | **Weight** |
| --- | --- | --- | --- | --- | --- | --- | --- |
| **Group** |  | **female** | **male** |  | **(months)** |  | **(kilograms)** |
| pVAX |  | 8 | 7 |  | 9.33 ± 0.98 |  | 153.46 ± 30.21 |
| Harvard |  | 8 | 7 |  | 9.27 ± 1.16 |  | 156.40 ± 30.73 |
| Aldevron |  | 9 | 6 |  | 9.53 ± 1.13 |  | 155.02 ± 27.32 |

**Supplemental Table 2.** Characteristics of buffalo in field trial 2.

|  |  | **Sex** | |  | **Age** |  | **Weight** |
| --- | --- | --- | --- | --- | --- | --- | --- |
| **Group** |  | **female** | **male** |  | **(months)** |  | **(kilograms)** |
| Mock  (extended) |  | 5 | 4 |  | 9.22 ± 0.79 |  | 119.37 ± 12.09 |
| pVAX  (extended) |  | 4 | 5 |  | 8.89 ± 0.82 |  | 120.58 ± 18.69 |
| SjC23-hsp70  (extended) |  | 6 | 5 |  | 8.96 ± 0.65 |  | 121.22 ± 24.39 |
| SjC23-hsp70  (regular) |  | 6 | 5 |  | 8.91 ± 0.66 |  | 121.45 ± 21.78 |

**Supplemental Table 3.** Characteristics of buffalo in field trial 3.

|  |  | **Sex** | |  | **Age** |  | **Weight** |
| --- | --- | --- | --- | --- | --- | --- | --- |
| **Group** |  | **female** | **male** |  | **(months)** |  | **(kilograms)** |
| pVAX |  | 4 | 5 |  | 9.25 ± 0.69 |  | 179.89 ± 12.83 |
| pSjC23 |  | 4 | 5 |  | 9.60 ± 0.86 |  | 180.56 ± 19.41 |
| pSjC23 + pIL-12 |  | 5 | 4 |  | 9.71 ± 0.88 |  | 188.00 ± 16.03 |

**Supplemental Table 4.** Characteristics of buffalo in field trial 4.

|  |  | **Sex** | |  | **Age** |  | **Weight** |
| --- | --- | --- | --- | --- | --- | --- | --- |
| **Group** |  | **female** | **male** |  | **(months)** |  | **(kilograms)** |
| pVAX  pVAX |  | 5 | 4 |  | 6.51 ± 0.56 |  | 108.78 ± 9.03 |
| pVAX  rSjC23 |  | 5 | 4 |  | 6.82 ± 0.68 |  | 103.78 ± 6.26 |
| pSjC23  rSjC23 |  | 4 | 5 |  | 6.48 ± 0.57 |  | 108.67 ± 8.50 |
| pSjC23/pSjTPI  rSj23/rSjTPI |  | 5 | 5 |  | 6.66 ± 0.61 |  | 108.90 ± 8.21 |
